# Bile Acids Impair Vaccine Response in Children With Biliary Atresia

**DOI:** 10.3389/fimmu.2021.642546

**Published:** 2021-04-16

**Authors:** Jinchuan Liu, Yi Fei, Tao Zhou, Hao Ji, Ji Wu, Xiangqian Gu, Yi Luo, Jianjun Zhu, Mingxuan Feng, Ping Wan, Bijun Qiu, Yefeng Lu, Tian Yang, Pengfei Deng, Cuiping Zhou, Dongcheng Gong, Jun Deng, Feng Xue, Qiang Xia

**Affiliations:** ^1^ Department of Liver Surgery and Liver Transplantation, Renji Hospital, School of Medicine, Shanghai Jiao Tong University, Shanghai, China; ^2^ Department of Immunology, Shanghai Pudong District Center for Disease Control and Prevention, Shanghai, China; ^3^ State Key Laboratory of Oncogenes and Related Genes, Shanghai Cancer Institute, and China-Australia Centre for Personalized Immunology, Renji Hospital, School of Medicine, Shanghai Jiao Tong University, Shanghai, China

**Keywords:** bile acids, vaccine response, children, biliary atresia, B cells

## Abstract

**Background:**

Vaccination is the best way to protect children under 5 years from death or disability. Children with biliary atresia (BA), which is the most common pediatric cholestatic end-stage liver disease (PELD), are more vulnerable to infectious diseases. However, the vaccination coverage and factors modulating vaccine responses in children with BA are largely unknown.

**Methods:**

In this study, 288 children (median age: 7 months) diagnosed with BA before liver transplantation were enrolled for the evaluation of vaccination status and the factors affecting the immune response to the hepatitis B (HBV) vaccine. Moreover, 49 BA children (median age: 4 months) were enrolled for flow cytometric analysis of CD4^+^ T cells and CD19^+^ B cell subsets and correlations with serum bile acid levels.

**Results:**

Generally, these children had very low routine vaccination rates for the meningococcal serogroup AC (Men AC) (41.2%), measles-mumps-rubella (MMR) (31.3%), poliomyelitis (Polio) (25.3%), hepatitis A (HAV) (25.0%), Japanese encephalitis (JE) (15.0%), diphtheria-tetanus-pertussis (DTP) (14.2%), meningococcal serogroup A (Men A) (13.5%) and varicella (VAR) (10.8%) vaccines, but not for the HBV (96.2%) and bacillus Calmette-Guérin (BCG) (84.7%) vaccines. Remarkably, 19.8% (57/288) of the patients had HBV infection. Out of 220 patients vaccinated for HBV, 113 (51.4%), 85 (38.6%) and 22 (10%) had one, two or three doses of the HBV vaccine, respectively. Furthermore, logistic regression analysis revealed that the bile acid level was an independent factor associated with poor HBV vaccine response (*p* = 0.03; OR = 0.394; 95% CI = 0.170-0.969). Immunophenotyping showed that bile acids were only negatively correlated with the CD19^+^CD27^+^IgG^+^ post-class-switched memory B cell ratio (*p* = 0.01).

**Conclusion:**

This study reveals the overall vaccination rates of routine vaccines in Chinese BA children are very low and the poor HBV vaccine responses are associated with bile acids, possibly *via* the inhibition of CD19^+^CD27^+^IgG^+^ post-class-switched memory B cell response.

**Clinical Trial Registration:**

http://www.chictr.org.cn, identifier ChiCTR1800019165.

## Introduction

Immunization is the most effective and economic way to prevent infection and disability and saves millions of children from death yearly (1, 2). It provides infants and young children protection against life-threatening infectious diseases such as influenza, polio, measles, meningitis and some cancers that may occur in adulthood (3–8). Chronic infection with hepatitis B virus (HBV) has a very high chance of developing into hepatocellular carcinoma and/or chronic liver failure (9), and timely and full-dose coverage of the HBV vaccine is the best way to prevent the development of hepatocellular carcinoma and liver failure (10). In China, the HBV vaccine is administered on a 3-dose schedule at 0, 1, and 6 months of age (11). The coverage of the three doses of this vaccine increased from 30% in 1992 to 99% in 2015 (12), and the incidence of HBV infection in children (≤5 years old) decreased from 10% in the 1990s to 0.3% in 2014 (13). Consequently, vaccination is the best protection against HBV infection in young children in China (≤5 years old) (14).

Pediatric cholestatic liver disease (PELD), characterized by progressive reduction or blockage of bile flow from the liver, often occurs in early life when liver function is immature and more vulnerable to endogenous or exogenous adverse consequences (15–18). Biliary atresia (BA) is the most common PELD characterized by progressive cholestasis (15) and is the leading cause of death in infants and young children, with an incidence of 1/5,000-1/20,000 worldwide (16, 19). The Kasai surgery and liver transplantation are currently applied to improve the survival rate and quality of life in infants with BA (16, 20, 21). One of the biggest challenges for BA patients is the high risk of HBV infection, given that 7-10% of the Chinese population is chronically infected with HBV (22), leading to a 1/4 risk of death from liver cancer or liver failure (23). Although timing and full-dose vaccination are critical before transplantation, the overall vaccination coverage of BA patients is generally poor for the following reasons: 1) delayed vaccination because of hospitalization; 2) parents and/or doctors who worry that vaccination might interfere with or worsen liver injury; and 3) BA patients who are too young (≤ 6 months) to receive 3 full doses of the vaccine (24, 25). Unfortunately, even with the same doses of the HBV vaccine, the antibody titers of BA children are significantly lower than those of age-matched healthy children (26), and the mechanism that impairs HBV vaccine responses in BA children is unclear.

Although vaccine-induced immune responses are highly variable among individuals, different populations and different regions of the world, several factors are recognized to modulate individual vaccination responses, including intrinsic factors (age, sex and genetics), extrinsic factors (infections and drugs), perinatal factors (gestational age and birth weight), environmental factors (geographic location and season), behavioral factors (anxiety and sleep) and nutritional factors (body mass index and enteropathy) (27–29). Bile acids, the major component of bile, are aberrantly elevated in children with BA (30). Several studies have identified a critical role of bile acids in regulating immune responses. For example, bile acids inhibit the proinflammatory function of macrophages, dendritic cells and natural killer T cells from innate immunity (31–33). In addition, bile acids suppress the differentiation of Th1 and Th17 cells but promote the differentiation of regulatory T cells (Tregs) (34, 35). However, the roles of bile acids in vaccine responses are largely unexplored.

In this study, we aimed to investigate 1) the overall coverage of routine vaccines in young Chinese BA patients and 2) whether bile acids have a role in the HBV vaccine response in young BA patients.

## Material and Methods

### Patient Recruitment

**Figure 1:**
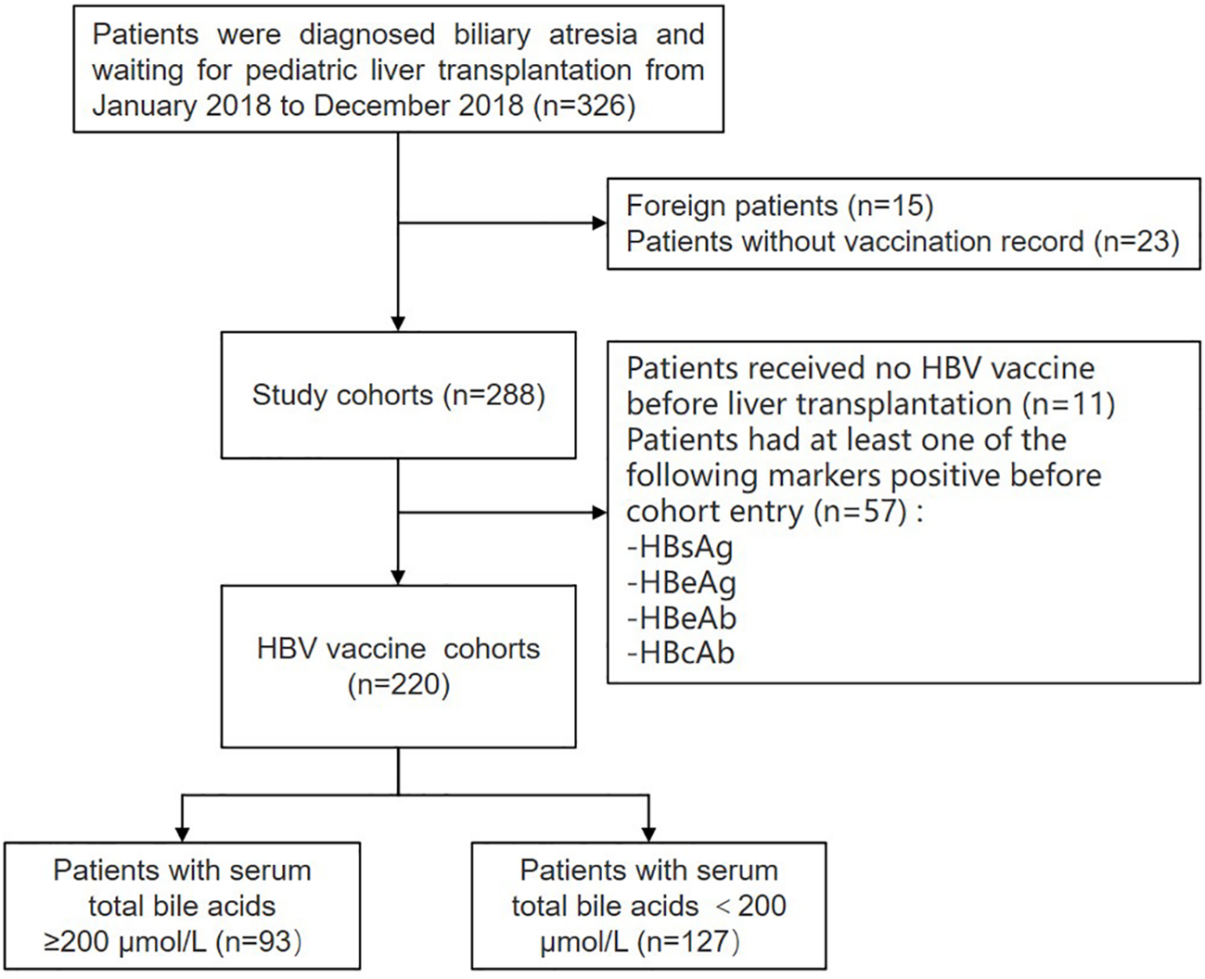
Flow chart: enrollment of patients with biliary atresia. HBV, hepatitis B virus; HBsAg, hepatitis B surface antigen; HBeAg, hepatitis B e-Antigen; HBeAb, hepatitis B e-antibody; HBcAb, hepatitis B core antibody.

A total of 326 patients with BA prior to liver transplantation were recruited from January 2018 to December 2018 at the Department of Liver Surgery of Renji Hospital, Shanghai Jiao Tong University School of Medicine (Shanghai, China). The exclusion criteria were as follows: foreign patients (*n* = 15) and those with missing vaccination records (*n* = 23). A total of 288 patients with detailed vaccination records were assessed. Eleven patients never received the HBV vaccine before liver transplantation, and 57 patients had at least one of the following markers positive before cohort entry: HBsAg/HBeAg/HBeAb/HBcAb (**Figure 1**). A total of 220 patients received at least one dose of the HBV vaccine. Their demographic data, clinical history, physical examination, and laboratory test results (i.e., HBsAb titers and bile acids levels) were all extracted from their medical records during the first visit.

For the flow cytometric analysis of CD4^+^ and B cell subsets in peripheral blood mononuclear cells (PBMCs), blood samples were collected from 49 BA children aged 4-21 months who met the inclusion criteria: (1) daily dose of glucocorticoid for children ≥ 10 kg not exceeding 20 mg/d; (2) daily dose of glucocorticoid for children weighing less than 10 kg not exceeding 2 mg/kg/d; and (3) the child had taken glucocorticoids exceeding the above dose for no more than two weeks.

### Serological Testing

HBV marker tests (HBsAg, HBsAb, HBeAg, HBeAb and HBcAb) in the serum of patients with BA before transplant were performed by ELISA (Genesis RMP150; Tecan Group, Zurich, Switzerland) following the manufacturer’s protocol. Children were classified as nonresponders or responders based on HBsAb levels: nonresponders (HBsAb levels < 10 mIU/mL), low/medium responders (HBsAb levels 10–200 mIU/mL), and high responders (HBsAb levels ≥ 200 mIU/mL).

### PBMC Isolation

Blood samples of 49 children with BA were collected in BD Vacutainer^®^ Blood Collection Tubes (BD). PBMCs were isolated by density gradient centrifugation with Ficoll-Paque (GE Healthcare) following the manufacturer’s protocol. In brief, blood samples were diluted with 0.9% NaCl (1:1) and gently loaded to the Ficoll layer, followed by density gradient centrifugation (500 g for 20 min at 20°C, acc/dec: 3/2). The mononuclear cell layer (intermediate white layer) was isolated, collected in a new tube, and rinsed with 0.9% NaCl. The cells were stored in liquid nitrogen or used for further assays.

### Flow Cytometry

Isolated PBMCs were incubated with the following fluorochrome-conjugated monoclonal antibodies: CD3 (HIT3a), CD4 (OKT4), CD19 (SJ25C1), CD20 (2H7), CD25 (BC96), CD27 (M-T271), CD38 (HIT2), CD45RA (HI100), CD127 (A019D5), CCR6 (G034E3), CXCR3 (G025H7), IgG (G18-145), IgM (CD38), and IgD (IA6-2). Dead cells were stained with Zombie Aqua (Biolegend). After staining at room temperature for 30 min, the cells were resuspended and examined with a FACS analyzer (Fortessa X-20, BD) and analyzed with FlowJo software (BD).

The flow cytometric gating strategy was slightly modified with respect to our previously reported protocol (36). CD4^+^ T cells (CD3^+^CD4^+^), CD19^+^ B cells (CD3^-^CD19^+^), Th1 cells (CD3^+^CD4^+^CXCR3^+^CCR6^-^), Th2 cells (CD3^+^CD4^+^CXCR3^-^CCR6^-^), Th17 cells (CD3^+^CD4^+^CXCR3^-^CCR6^+^), Treg cells (CD3^+^CD4^+^CD25^+^CD127^-^), CD19^+^CD27^+^IgG^+^ post-class-switched memory B cells, naive B cells (CD3^-^CD19^+^IgD^+^CD27^-^), class-switched memory B cells (CD3^-^CD19^+^IgD^-^CD27^+^), non-class-switched memory B cells (CD3^-^CD19^+^IgD^+^CD27^+^), double-negative memory B cells (CD3^-^CD19^+^IgD^-^CD27^-^) and plasma B cells (CD3^-^CD19^+^IgD^-^CD27^+^IgM^-^CD20^-^CD38^+^).

### Data Analysis

All data were analyzed using SPSS (version 22) or GraphPad Prism (version 8). Continuous variables are displayed as medians (IQRs) and analyzed using two-sample t tests or Wilcoxon rank-sum tests; categorical variables are determined as percentages and analyzed using Fisher’s exact test. Multiple logistic regression models were used to calculate ORs and corresponding 95% CIs to analyze the associations between patients with TBA ≥ 200 μmol/L and TBA < 200 μmol/L, adjusting for dose of vaccination, weight, height, Kasai surgery, TBIL and CHOL. All statistical tests were 2-tailed, and a p-value < 0.05 was considered statistically significant.

### Study Approval

The study was approved by the Ethical Committee of Renji Hospital, School of Medicine, Shanghai Jiao Tong University [(2017)209]. All patients were provided with written informed consent.

## Results

### Low Vaccination Coverage for Routine Vaccines and High Incidence of HBV Infection in Children With BA

A total of 326 patients with BA prior to liver transplantation were recruited from January 2018 to December 2018 at the Department of Liver Surgery of Renji Hospital, Shanghai Jiao Tong University School of Medicine (Shanghai, China). Fifteen foreign patients and 23 patients without vaccination records were excluded. A total of 288 patients with detailed vaccination records were assessed. The median age was 7 months (range: 4 months - 159 months). From birth to liver transplantation, 277 (96.2%) patients received at least one dose of the HBV vaccine. The vaccination rates for other vaccines included in the China National Immunization Program were also analyzed. The vaccination rates for the HBV (96.2%, 277/288) and bacillus Calmette-Guérin (BCG) (84.7%, 244/288) were much higher than the rates of meningococcal serogroup A/C (Men AC) (41.2%, 7/17), measles/mumps/rubella (MMR) vaccine (31.3%, 15/48), poliomyelitis (polio) (25.3%, 73/288), hepatitis A (HAV) (25.0%, 12/48), Japanese encephalitis (JE) (15.0%, 18/120), diphtheria/tetanus/pertussis (DTP) (14.2%, 41/288), meningococcal serogroup A (Men A) (13.5%, 29/215), and varicella (VAR) vaccines (10.8%; 7/65) (**Table 1**). These results show that vaccination coverage in young patients with BA prior to liver transplantation are very low compare to the overall vaccination coverage of over 95% in China (37).

**Table 1:** The overview of the vaccinations of the enrolled BA patients.

Vaccine	Total (n=288)	Time recommended for first dose of vaccination after birth (month)
Age (month), median (range)	7 (4-159)	/
Female, n (%)	167 (58.0)	/
HBV	277/288 (96.2%)	0
BCG	244/288 (84.7%)	0
Polio	73/288 (25.3%)	2
DTP	41/288 (14.2%)	3
Men A	29/215 (13.5%)	6
JE	18/120 (15.0%)	8
VAR	7/65 (10.8%)	12
MMR	15/48 (31.3%)	18
HAV	12/48 (25.0%)	18
Men AC	7/17 (41.2%)	36

BCG, bacillus Calmette–Guérin vaccine; DTP, diphtheria/tetanus/pertussis vaccine; HAV, hepatitis A vaccine; HBV, hepatitis B vaccine; JE, Japanese encephalitis vaccine; Men A, meningococcal serogroup A vaccine; Men AC, meningococcal serogroup A/C vaccine; MMR: measles/mumps/rubella vaccine; Polio, poliomyelitis vaccine; VAR, varicella vaccine.

Given that one of the greatest risks for young children with BA is HBV infection, we focused on the analysis of HBV vaccination coverage in the BA pediatric population. Among 288 patients with vaccination records, 11 (3.8%) did not receive the timely birth-dose vaccination, and 57 (19.8%) patients tested positive for at least one of the following markers: hepatitis B surface antigen (HBsAg)/hepatitis B e antigen (HBeAg)/hepatitis B e antibody (HBeAb)/hepatitis B core antibody (HBcAb), revealing a very high HBV infection rate (19.8%). Even more importantly, the level of HBV was twofold higher than the one previously reported from the biopsy of tissues in infants with biliary atresia (HBV infection rate was 9%) (38).

### Low Completion Rate of the HBV Vaccine and Defective Antibody Response

The median age of the 220 patients who received at least one dose of the vaccine was 7 months (range: 4 months - 131 months); 84 (38.2%) were male, and 136 (61.8%) were female. A total of 113 (51.4%) patients received one dose of the HBV vaccine, 85 (38.6%) received two doses, and only 22 (10%) completed three doses of the HBV vaccine according to the China’s National Immunization Program (**Table 2**), demonstrating that the full coverage of the HBV vaccine in young patients with BA is extremely low. Seventy-five (34.1%) patients were nonresponders (HBsAb titer lower than 10 mIU/mL), 94 (42.7%) patients were low/medium responders (HBsAb titer between 10 and 200 mIU/mL), and 51 (23.2%) patients were high responders (HBsAb higher than 200 mIU/mL). The proportion of high responders was significantly increased in patients received two or three doses of the vaccine (**Table 3**). Taken together, these results demonstrate the low rate of full-dose HBV vaccine coverage and a low ratio of high responders in young children with BA.

**Table 2:** Factors affecting immune response to HBV vaccine.

Characteristics	All (n=220)	HBsAb < 200 (mIU/mL) (n=51)	HBsAb ≥ 200 (mIU/mL) (n=169)	*p*-value
Age, median (IQR)	7 (6-11)	7 (6-11)	7 (5-14)	0.62
Female/male, n	136/84	34/17	102/67	0.42
Weight (kg), median (IQR)	7.4 (6.5-8.8)	7.4 (6.5-8.8)	7.3 (6.5-9.1)	0.75
Weight (%), median (IQR)	9.0 (1.4-31.0)	8.00 (1.0-30.0)	12.4 (2.7-33.0)	0.42
Height (kg), median (IQR)	66 (64-72)	66 (64-71)	66 (64-75)	0.83
Height (%), median (IQR)	10.3 (0.8-27.6)	11.3 (0.8-27.7)	9.2 (1.8-22.9)	0.90
BMI (kg/m^2^), median (IQR)	16.6 (15.3-17.8)	16.4 (15.3-17.8)	16.7 (15.3-17.9)	0.73
**Biochemical parameters median** (IQR)				
Albumin (g/L)	34.1 (30.2-38.2)	34.0 (30.2-38.2)	34.20 (30.7-40.1)	0.40
ALT (U/L)	127.0 (82.3-210.0)	128.0 (82.0-210.0)	125.0 (83.0-223.0)	0.98
AST (U/L)	218.5 (151.5-361.3)	216.0 (50.5-343.0)	230.0 157.0-396.0)	0.60
GGT (U/L)	273.0 (127.3-523.0)	277.0 (127.5-589.2)	244.8 (118.0-497.0)	0.25
ALP (U/L)	585.0 (423.8-781.0)	570.0 (396-773.5)	622.0 (518-813)	0.16
TBA (µmol/L)	175.5 (134.0-271.9)	188.8 (141.8-276)	144.4 (97.8-199.5)	<0.01
TBIL (µmol/L)	241.3 (123.4-338.0)	233.3 (137.6-338.3)	255.3 (51.5-352.1)	0.74
CHOL (mmol/L)	4.8 (3.6-6.7)	4.7 (3.5-6.6)	4.9 (3.6-6.8)	0.76
White blood cells (x10^9^/L)	10.6 (8.0-13.5)	10.7 (8.2-13.6)	9.6 (7.1-12.6)	0.25
Neutrophils (x10^9^/L)	3.9 (2.6-5.5)	4.0 (2.8-5.9)	3.4 (2.4-5.2)	0.18
Lymphocytes (x10^9^/L)	4.8 (3.3-7.0)	4.9 (3.6-7.0)	4.6 (3.1-7.1)	0.66
Monocytes (x10^9^/L)	0.8 (0.6-1.2)	0.9 (0.6-1.2)	0.7 (0.5-1.1)	0.07
Kasai surgery, n (%)				
Yes	70 (31.8%)	18 (35.3%)	52 (30.8%)	0.54
No	150 (68.2%)	33 (64.7%)	117 (69.2%)	
Dose of vaccination, n (%)				
One	113 (51.4%)	5 (9.8%)	108 (63.9%)	<0.01
Two	85 (38.6%)	34 (66.7%)	51 (30.2%)	
Three	22 (10%)	12 (23.5%)	10 (5.9%)	
Time since vaccination (month), median (IQR)	6.0 (4.3-9.0)	4.0 (3.0-7.0)	7.0 (5.0-10.0)	<0.01

The p-value stands for the statistical results between HBsAb < 200 (mIU/mL) and HBsAb ≥ 200 (mIU/mL). Values are summarized as median (IQR). Categorical variables are summarized as percentages. P for statistical significance was obtained using Fisher’s exact test for categorical variables or independent t-test or Mann-Whitney U test for continuous variables, as appropriate. IQR, interquartile range.

**Table 3:** Definition of non, low/medium and high responders based on HBsAb titers and coverage.

Vaccine dose	Nonresponders (n=75)	Low/medium responders (n=94)	High responders (n=51)
One (n=113)	57 (50.4%)	51 (45.1%)	5 (4.4%)
Two (n=85)	11 (12.9%)	40 (47.1%)	34 (40.0%)
Three (n=22)	7 (31.8%)	3 (13.6%)	12 (54.5%)

Nonresponders: HBsAb < 10 mIU/mL.

Low/medium responders: HBsAb 10–200 mIU/mL.

High responders: HBsAb levels ≥ 200 mIU/mL.

### Serum Bile Acids Are Decreased in Responders With High HBsAb Titers

**Figure 2:**
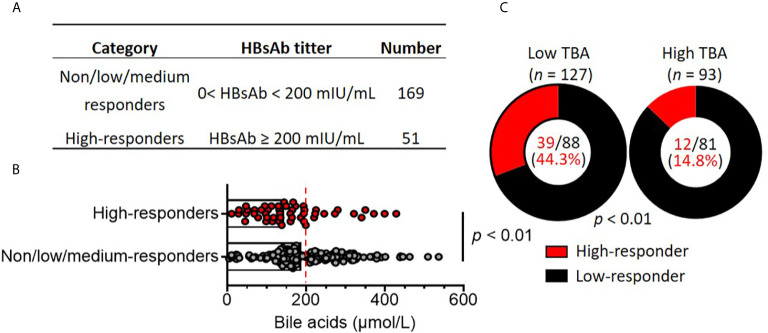
High bile acid levels are associated with poor HBV vaccine responses in patients with biliary atresia. **(A)** Children with biliary atresia (*n =* 220) were classified as non, low, medium (no/low/medium, Low) responders and high responders based on HBsAb titers after vaccination with the HBV vaccine. **(B)** Comparison of serum bile acid levels between non/low/medium responders (*n =* 169) and high responders (*n =* 51). The dotted line indicates subjects with low bile acid levels (0-200 mmol/L) and high bile acid levels (≥ 200 mmol/L). **(C)** Comparison of responder rates between the low bile acid (*n =* 127) and high bile acid (*n =* 57) groups.

Various factors modulate the immune response to vaccination (27). To investigate the potential factors that may impact the antibody responses to the HBV vaccine, the patients were divided into two groups based on HBsAb titers: non/low/medium responders (HBsAb < 200 mIU/mL, n = 51) and high responders (≥ 200 mIU/mL, n = 169) (**Figure 2A**). Fisher’s exact test assay was applied to compare parameters, including body weight, height, and clinical parameters. There were no differences between the two groups according to demographic parameters, such as age, sex, weight, height and BMI. Next, laboratory biochemical parameters were compared. Levels of albumin (ALB), alanine aminotransferase (ALT), aspartate transaminase (AST), gamma-glutamyl transferase (GGT), alkaline phosphatase (ALP), total bilirubin (TBIL), cholesterol (CHOL) and white blood cell, neutrophil, lymphocyte and monocyte counts were similar between the non/low/medium and high responders (**Table 2**). Surprisingly, total bile acid (TBA) levels were dramatically reduced in the high responders compared to the non/low/medium responders, with a nearly 25% reduction (median: 144.4 µmol/L vs 188.8 µmol/L) (*p* < 0.01) (**Figure 2B**). The history of the Kasai surgery, vaccination dosage and time since vaccination were also compared between the two groups. We found that vaccination dosage and time since vaccination were significantly different between the non/low/medium and high responders (**Table 2**). Altogether, these assays reveal that serum bile acids are decreased in responders with high HBsAb titers.

To further analyze the association of serum bile acid levels with HBsAb titers in non/low/medium and high responders, the patients were subdivided into low (TBA < 200 µmol/L) and high total serum bile acids (TBA ≥ 200 µmol/L). There were 127 (57.7%) patients with serum TBA < 200 µmol/L, with a median value of 139.3 μmol/L. Ninety-three patients had TBA ≥ 200 µmol/L (median value: 279.3 μmol/L), which was 2-fold higher than the patients with low serum TBA. There were no significant differences between the high and low serum TBA levels according to demographic parameters, such as age, sex, weight, height and BMI. In addition, the laboratory biochemical parameters ALB, ALT, AST, GGT, ALP, TBIL, white blood cells, neutrophils, lymphocytes and monocytes in patients with low and high serum TBA were similar. Remarkably, only CHOL levels in the low TBA patients (median: 4.3 mmol/L) were significantly lower than those in the high TBA patients (median: 5.9 mmol/L) (*p* < 0.01) (**Table 4**).

**Table 4:** Characteristics of patients grouped by TBA.

Characteristics	TBA< 200 µmol/L (n=127)	TBA≥ 200 µmol/L (n=93)	*p*-value
Age, median (IQR)	7 (5-14)	7 (6-10)	0.54
Female/male, n	52/75	32/61	0.40
Weight (kg), median (IQR)	7.4 (6.5-9.5)	7.3 (6.5-8.0)	0.08
Weight (percentage), median (IQR)	10.4 (1.7-34.9)	6.9 (1.1-28.1)	0.20
Height (kg), median (IQR)	66 (64-75)	66 (64-70)	0.09
Height (percentage), median (IQR)	12.1 (1.2-28.5)	7.9 (0.6-21.4)	0.39
BMI (kg/m^2^), median (IQR)	16.7 (15.4-17.8)	16.2 (15.2-17.6)	0.40
**Biochemical parameters,** median (IQR)			
Albumin (g/L)	34.0 (28.9-38.3)	34.3 (31.4-38.0)	0.47
ALT (U/L)	121.0 (82.0-186.0)	132.0 (82.0-230.5)	0.45
AST (U/L)	219.0 (139.0-386.0)	215.0 (156.5-344.5)	0.56
GGT (U/L)	244.8 (118.0-523.0)	324.0 (158.0-556.0)	0.13
ALP (U/L)	577.0 (413.0-783.0)	590.0 (456.0-781.0)	0.42
TBIL (µmol/L)	223.1 (47.2-341.1)	258.2 (168.0-336.9)	0.05^#^
CHOL (mmol/L)	4.3 (3.1-6.2)	5.9 (3.9-9.0)	<0.01
White blood cells (x10^9^/L)	9.7 (7.2-13.3)	11.4 (8.3-14.3)	0.06
Neutrophils (x10^9^/L)	3.7 (2.6-5.3)	4.2 (2.8-6.3)	0.27
Lymphocytes (x10^9^/L)	4.5 (3.1-7.0)	5.4 (3.7-7.2)	0.08
Monocytes (x10^9^/L)	0.8 (0.5-1.2)	0.9 (0.6-1.2)	0.28
**Kasai surgery, n (%)**			0.08
Yes	34 (26.8%)	36 (38.7%)	
No	93 (73.2%)	57 (61.3%)	
**Dose of vaccination, n (%)**			0.01
One	55 (43.3%)	58 (62.4%)	
Two	54 (42.5%)	31 (33.3%)	
Three	18 (14.2%)	4 (4.3%)	
Time since vaccination (month), median (IQR)	6.0 (4.0-10.0)	6.0 (5.0-8.5)	0.40

P values are summarized as median (IQR). Categorical variables are summarized as percentages. P for statistical significance was obtained using Fisher’s exact test for categorical variables or independent t-test or Mann-Whitney U test for continuous variables, as appropriate. IQR, interquartile range; ^#^p=0.0535.

Among the 127 patients with low serum TBA, 88 were non/low/medium responders with HBsAb titers < 200 mIU/mL (median: 12.7 mIU/mL). Only 39 patients were high responders (median: 830.2 mIU/mL). Among the 93 patients with high serum TBA, 81 were non/low/medium responders with HBsAb titers < 200 mIU/mL (median: 16.67 mIU/mL), and only 12 patients were high responders (median: 333.2 mIU/mL). The ratio of high responders/non/low/medium responders (39/88) in low serum TBA patients was 3.0-fold higher than the ratio of high responders/non/low/medium responders (12/81) in high TBA patients (**Figure 2C**). Logistic regression analysis after adjustment for covariates (weight, height, vaccine dose, Kasai surgery, TBIL and CHOL) (**Table 4**) showed that only high serum TBA levels were associated with non/low/medium responders to the HBV vaccine (OR = 0.394; 95% CI = 0.170-0.969, *p* = 0.03) (**Table 5**). In summary, the patients with high serum bile acid levels were associated with poor HBV vaccine responses.

**Table 5:** Multivariate logistic regression analysis of factors affecting the immune response to the HBV vaccine between the high and low TBA groups.

	Adjusted OR (95%CI)	*p*-value
Bile acids	0.394 (0.170-0.969)	0.03
Vaccine dose		
One		
Two	15.440 (5.385-44.271)	<0.01
Three	164.579 (22.579-1199.647)	<0.01
Weight	1.024 (0.713-1.470)	0.90
Height	0.927 (0.829-1.037)	0.19
Kasai surgery	1.290 (0.552-3.018)	0.56
TBil	1.001 (1.000-1.002)	0.17
Chol	1.062 (0.967-1.167)	0.21

The p-value for statistical significance was obtained using multivariate logistic regression.

### Serum Bile Acid Levels Are Negatively Correlated With Post-Class-Switched Memory B Cells but Not CD4^+^ T Cells in BA Patients

**Figure 3:**
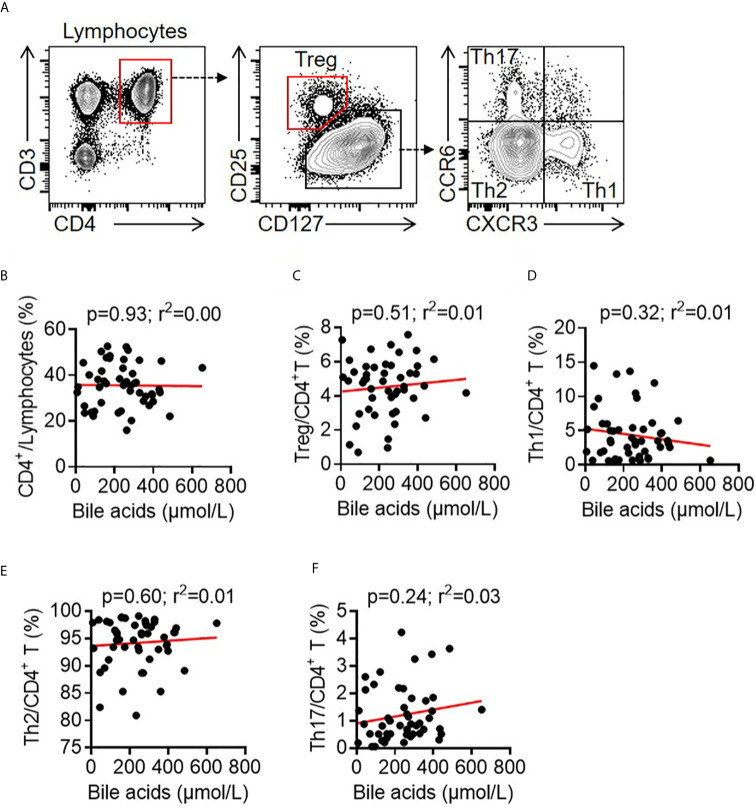
Correlation between bile acids and T cells in BA children. **(A)** Representative FACS dots of CD4^+^ T (CD3^+^CD4^+^), Treg cells (CD3^+^CD4^+^CD25^+^CD127^-^), Th1 cells (CD3^+^CD4^+^CXCR3^+^CCR6^-^), Th2 cells (CD3^+^CD4^+^CXCR3^-^CCR6^-^), and Th17 cells (CD3^+^CD4^+^CXCR3^-^CCR6^+^) in the PBMCs of patients with BA. **(B–F)** Correlation between bile acids and CD4^+^ T cells, Treg cells, Th1 cells, Th2 cells and Th17 cells in the PBMCs of patients with BA.

Bile acids play vital roles in regulating immune responses, such as natural killer cells, macrophages, dendritic cells, B cells and CD4^+^ T cells (31, 34, 35, 39). CD4^+^ T cells contribute to the antibody response during vaccination, and bile acids selectively promote regulatory T cells and inhibit Th17 cells and IL-22-producing cells (34, 40). To analyze CD4^+^ T cell profiling in young children with BA, 49 young patients with BA were recruited. Circulating CD4^+^ T cell subsets in human peripheral blood mononuclear cells (PBMCs) were analyzed by FACS. Contrary to previous findings that bile acids selectively modulate Treg and Th17 cell responses (34), there were no correlations between CD4^+^ T cell ratios (*r^2^* = 0.00, *p* = 0.93), Treg (*r^2^* = 0.01, *p* = 0.51), Th1 (*r^2^* = 0.01, *p* = 0.32), Th2 (*r^2^* = 0.01, *p* = 0.60), Th17 cells (*r^2^* = 0.03, *p* = 0.24) and serum total bile acid levels in young children with BA (**Figure 3**).

**Figure 4:**
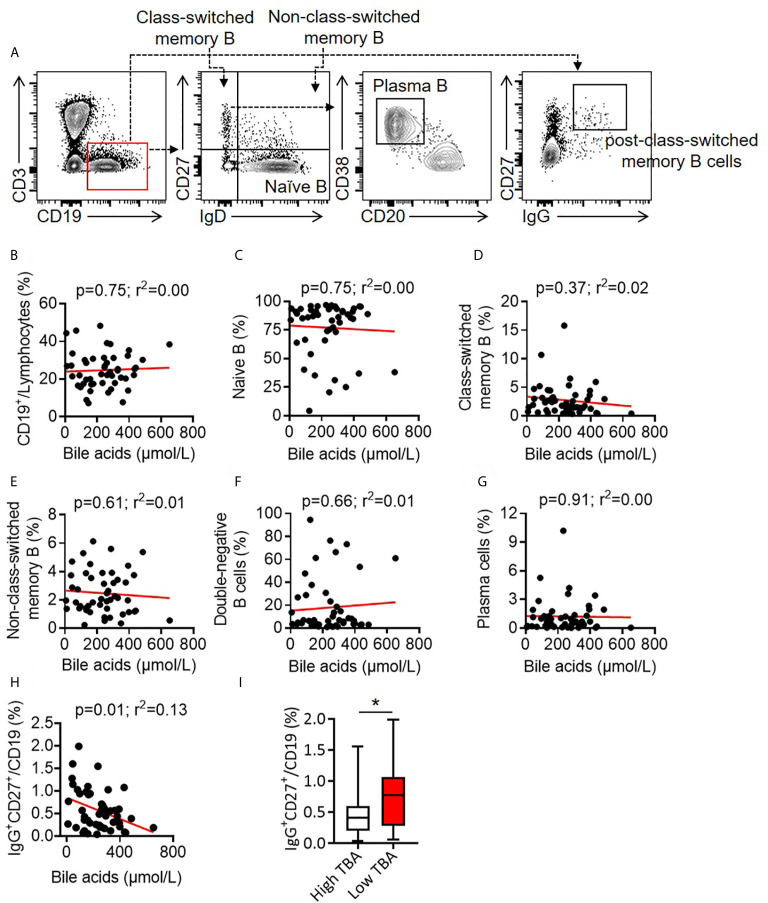
Correlation between bile acid levels and B cells in BA children. **(A)** Representative FACS dots of B cells (CD3^-^CD19^+^), naive B cells (CD3^-^CD19^+^IgD^+^CD27^-^), class-switched memory B cells (CD3^-^CD19^+^IgD^-^CD27^+^), non-class-switched memory B cells (CD3^-^CD19^+^IgD^+^CD27^+^), double-negative memory B cells (CD3^-^CD19^+^IgD^-^CD27^-^), post-class-switched memory B cells (CD19^+^CD27^+^IgG^+^) and plasma cells (CD3^-^CD19^+^IgD^-^CD27^+^IgM^-^CD20^-^CD38^+^) in the PBMCs of patients with BA. **(B–H)** Correlation between bile acid levels and B cells **(B)**, naive B cells **(C)**, class-switched memory B cells **(D)**, non-class-switched memory B cells **(E)**, double-negative memory B cells **(F)**, plasma cells **(G)** and post-class-switched memory B cells **(H)** in the PBMCs of patients with BA. **(I)** Proportion of CD19^+^CD27^+^IgG^+^ post-class-switched memory B cells between high TBA and low TBA groups * means *p < 0.05*.

B cells are antibody-producing cells and play crucial roles in vaccine efficacy (41, 42). There was no correlation between total CD3-CD19^+^ B cells and total BA (*r^2^* = 0.00, *p* = 0.75). TBA levels did not correlate with naive B cells (*r^2^* = 0.00, *p* = 0.75), class-switched memory B cells (*r^2^* = 0.02, *p* = 0.37), non-class-switched memory B cells (*r^2^* = 0.01, *p* = 0.61), double-negative memory B cells (*r^2^* = 0.01, *p* = 0.66) or plasma cells (*r^2^* = 0.00, *p* = 0.91) (**Figures 4A–G**). Notably, TBA levels were negatively correlated with CD19^+^CD27^+^IgG^+^ post-class-switched memory B cells (*r^2^* = 0.13, *p* = 0.01) (**Figure 4H**) and the median percentage of CD19^+^CD27^+^IgG^+^ cells in Low TBA group was 0.77%, which was much higher than 0.42% in High TBA group (**Figure 4I**), indicating that bile acids selectively regulate B cell subsets, specifically inhibiting post-class-switched memory B cells. These evidences show that serum BA levels are negatively correlated with CD19^+^CD27^+^IgG^+^ post-class-switched memory B cells.

## Discussion

Children under 5 years old are at high risk of developing life-threatening infectious diseases. The timely and full coverage of vaccines saves millions of lives each year (1). BA, a cholestatic end-stage liver disease, is the leading cause of death among liver diseases in early life. However, in even more vulnerable infants/young children with BA, information on the overall coverage of HBV and other vaccines is poor and elusive. Our study first reported vaccination coverage for 10 types of vaccines in 288 patients with BA in China. Our results showed very low coverage (10.8% - 41.2%) for the polio, DTP, MMR, JE, Men A, Men AC, VAR and HAV vaccines. The incidence of HBV infection was 19.7%, which was much higher than that previously reported (22). In addition, among the 220 patients with the HBV vaccine, 113 (51.4%) received one dose of the HBV vaccine, 85 (38.6%) received 2 doses, and only 22 (10%) completed three doses of the HBV vaccine. Our study might be the first to report the low completion rate of the HBV vaccine in Chinese children with BA. Moreover, we found that high serum bile acids were associated with defective HBV vaccine responses and that bile acids were negatively correlated with CD19^+^CD27^+^IgG^+^ post-class-switched memory B cells.

The Department of Liver Surgery and Liver Transplantation of Renji Hospital is the largest single center for pediatric liver transplantation in China, with over 400 patients per year, and 70% of them are under 3 years old. From January 2018 to December 2018, 288 BA patients waiting for liver transplantation were enrolled in this study. Among them, 57 (19.7%) patients tested positive for the HBV infection, which is much higher than previously reported (22). These data probably represent the very first report on HBV infection in young Chinese patients with BA before liver transplantation.

Full-dose coverage of the HBV vaccine has successfully reduced the incidence of HBV infection in children under 5 years old in China (13). Even with the same doses of HBV vaccine, BA patients have much lower HBsAb titers than age- and sex-matched healthy children (26). Among 220 patients with HBV vaccine (≥ 1 dose), 75 (34.1%) were nonresponders with HBsAb (< 10 mIU/mL), 94 (42.7%) were low/medium responders with HBsAb (10-200 mIU/mL) and 51 (23.2%) patients were high responders with HBsAb (≥ 200 mIU/mL). These findings are consistent with previous reports on BA children with a higher percentage of non-responders (26, 43), indicating that intrinsic factors may impact antibody production in children with BA. Various factors, such as nutrition, drug usage, and environmental and genetic factors contribute to the variability of individual responses to vaccines (27, 28). To screen potential factors that regulate the HBV vaccine response, patients were divided into high responders and non/low/medium responders based on HBsAb titers. In addition to the dose of vaccine and time since vaccination are known to affect vaccine response (27), the serum bile acid levels were significantly lower in high responders than in non/low/medium-responders (median bile acid levels: 144.4 vs 188.8 µmol/L, *p* < 0.01). Likewise, the division of patients in the low serum bile acids group (*n* = 127) and high serum bile acids group (*n* = 93) resulted in 39 high responders out of 127 patients with low serum bile acids, whereas only 12 high responders out of 93 patients had high serum bile acids, finally showing a 2.4-fold reduction (39/127: 12/93). The logistic regression analysis after adjustment for covariates showed that high serum TBA was associated with non/low/medium responders to the HBV vaccine (*OR* = 0.394; *95% CI* = 0.170-0.969, *p* = 0.03). These evidences indicate that high serum bile acids are associated with poor HBV vaccine response in children with BA.

CD4^+^ T cells and B cells are critical for antigen-specific antibody production in vaccination (42, 44). We compared the serum bile acid levels with CD4^+^ T cell subsets and B cells in newly recruited BA patients. There were no correlations of serum bile acid levels with the ratios of total CD4^+^ T cells, Treg cells, Th1, Th2 or Th17 cells. Contrary to previous findings that bile acids promote Treg cells and inhibit Th17 cells in adult patients (34), these results may be due to the immaturity of the immune systems in young children investigated in our study (median: 5 months). Moreover, there was no correlation of serum bile acids with total CD19^+^ B cells, naïve B cells, class-switched memory B cells, double-negative memory B cells, or non-class-switched memory B cells, but a significant correlation was found with CD19^+^CD27^+^IgG^+^ post-class-switched memory B cells (*r^2^* = 0.13, *p* = 0.01). According to our knowledge, there is only one report stating that PBMCs with 5-day stimulation with Staphylococcus aureus Cowan I (SAC-I) and the addition of hydrophobic bile acids (chenodeoxycholic acid, CDCA; ursodeoxycholic acid, UDCA) may strongly depress IgM production but not IgA or IgG *in vitro* culture (45).

Metabolites of bile acids regulate the balance of Treg cells and Th17 cells in intestinal inflammation (34) and reduce both humoral and cellular immunity in patients with primary biliary cholangitis (PBC) (46–48), however, the modulatory roles of bile acids in vaccination responses either in adults or children have not been reported so far. Our ongoing projects is trying to delineate the potential mechanism of bile acids in reducing humoral immunity in children with BA. This study indicates that bile acids are significantly correlated with CD19^+^CD27^+^IgG^+^ post-class-switched memory B cells in children with BA. However, the molecular mechanism of bile acids on B cell responses during HBV vaccination needs further investigation.

The current lack of understanding or knowledge concerning the importance of vaccination dosage and timing by doctors and/or parents is at the basis of the remarkably low (10%, 22/220) completion rate of the HBV vaccine in BA patients in China. More importantly, this might account for the high incidence of HBV infection (19.8%, 57/288). Therefore, we highly recommend that doctors and parents of BA patients gradually realize the importance of completing the full-dose of the HBV vaccine.

In conclusion, our findings shed light on the low coverage of routine vaccines and the high incidence of HBV infection in China. Moreover, our study adds new scientific evidence by demonstrating that high serum bile acid levels are associated with poor HBV vaccine responses in BA patients, potentially *via* the modulatory roles of TBA on CD19^+^CD27^+^IgG^+^ post-class-switched memory B cells.

## Data Availability Statement

The original contributions presented in the study are included in the article/supplementary material. Further inquiries can be directed to the corresponding authors.

## Ethics Statement

The studies involving human participants were reviewed and approved by Ethical Committee of Renji Hospital, School of Medicine, Shanghai Jiao Tong University. Written informed consent to participate in this study was provided by the participants’ legal guardian/next of kin.

## Author Contributions

Study conception and design: FX and QX. Vaccination data collection: YF, TY, PD, CZ. Clinical data collection: HJ, TZ, JW, XG, JZ, MF, PW, BQ, YL, YFL, DG. Experiments performing and manuscript writing: JL, JD and FX. All authors contributed to the article and approved the submitted version.

## Funding

This project was supported by the National Natural Science Foundation of China (81670602 to FX, 82071816 to JD), Shanghai Municipal Hospital Three-year-project for Clinical Skills’ Promotion and Innovation (16CR1003A), and Renji Hospital Clinical Research Innovation Incubation Fund Plan (PYI-17-002), School of Medicine, Shanghai Jiao Tong University.

## Conflict of Interest

The authors declare that the research was conducted in the absence of any commercial or financial relationships that could be construed as a potential conflict of interest.
